# Insights into Genetic Susceptibility to Melanoma by Gene Panel Testing: Potential Pathogenic Variants in *ACD*, *ATM*, *BAP1*, and *POT1*

**DOI:** 10.3390/cancers12041007

**Published:** 2020-04-19

**Authors:** Lorenza Pastorino, Virginia Andreotti, Bruna Dalmasso, Irene Vanni, Giulia Ciccarese, Mario Mandalà, Giuseppe Spadola, Maria Antonietta Pizzichetta, Giovanni Ponti, Maria Grazia Tibiletti, Elena Sala, Maurizio Genuardi, Pietro Chiurazzi, Gabriele Maccanti, Siranoush Manoukian, Serena Sestini, Rita Danesi, Valentina Zampiga, Roberta La Starza, Ignazio Stanganelli, Alberto Ballestrero, Luca Mastracci, Federica Grillo, Stefania Sciallero, Federica Cecchi, Enrica Teresa Tanda, Francesco Spagnolo, Paola Queirolo, Italian Melanoma Intergroup (IMI), Alisa M. Goldstein, William Bruno, Paola Ghiorzo

**Affiliations:** 1 Genetics of Rare Cancers, Department of Internal Medicine and Medical Specialties, University of Genoa, 16132 Genova, Italy; l.pastorino@unige.it (L.P.); virginia.andreotti@unige.it (V.A.); brunasamia.dalmasso@dimi.unige.it (B.D.); irene.vanni@hsanmartino.it (I.V.); giuliaciccarese@libero.it (G.C.); william.bruno@unige.it (W.B.); 2 IRCCS Ospedale Policlinico San Martino, 16132 Genova, Italy; aballestrero@unige.it (A.B.); luca.mastracci@unige.it (L.M.); federica.grillo@unige.it (F.G.); 3 Unit of Medical Oncology, Department of Oncology and Hematology, Papa Giovanni XXIII Hospital, 24127 Bergamo, Italy; mmandala@asst-pg23.it; 4 Divisione di Chirurgia del Melanoma, IRCCS Fondazione Istituto Nazionale per lo studio e la cura dei tumori, 20133 Milano, Italy; giuseppe.spadola@istitutotumori.mi.it; 5 Dermatologic Clinic, University of Trieste, 34127 Trieste, Italy; pizzichetta@cro.it; 6 Department of Medical Oncology, Centro di Riferimento Oncologico di Aviano (CRO) IRCCS, 33081 Aviano, Italy; 7 Department of Diagnostic and clinical medicine and public health, University of Modena and Reggio Emilia, 41124 Modena, Italy; giovanni.ponti@unimore.it; 8 Department of Pathology, ASST Sette Laghi, 21100 Varese, Italy; mariagrazia.tibiletti@ospedale.varese.it; 9 Medical Genetics Laboratory, Clinical Pathology Department, S. Gerardo Hospital, 20900 Monza, Italy; elena.sala@asst-monza.it; 10 UOC Genetica Medica, Fondazione Policlinico Universitario A. Gemelli IRCCS, 00168 Roma, Italy; Maurizio.Genuardi@unicatt.it (M.G.); pietro.chiurazzi@unicatt.it (P.C.); 11 Sezione Genetica Medica, Dipartimento di Scienze della Vita e di Sanità Pubblica, Università Cattolica del Sacro Cuore, 00168 Roma, Italy; 12 UO Dermatologia P.O. Misericordia, 58100 Grosseto, Italy; maccanti@aliceposta.it; 13 Unit of Medical Genetics, Department of Medical Oncology and Hematology, Fondazione IRCCS Istituto Nazionale dei Tumori di Milano, 20133 Milano, Italy; Siranoush.Manoukian@istitutotumori.mi.it; 14 Plastic & Reconstructive Surgery Unit, Regional Melanoma Referral Center and Melanoma & Skin Cancer Unit Tuscan Tumour Institute (ITT), Santa Maria Annunziata Hospital, 50012 Firenze, Italy; serena.sestini@uslcentro.toscana.it; 15 Romagna Cancer Registry, IRCCS-IRST Scientific Institute of Romagna for the Study and Treatment of Cancer, 47014 Meldola, Italy; rita.danesi@irst.emr.it; 16 Biosciences Laboratory, Istituto Scientifico Romagnolo per lo Studio e la Cura dei Tumori (IRST) IRCCS, 47014 Meldola, Italy; valentina.zampiga@irst.emr.it; 17 Hematology and Bone Marrow Transplantation Unit, CREO, University of Perugia, 06156 Perugia, Italy; roberta.lastarza@unipg.it; 18 Skin Cancer Unit, IRCCS-IRST Scientific Institute of Romagna for the Study and Treatment of Cancer, 47014 Meldola, Italy; ignazio.stanganelli@irst.emr.it; 19 Department of Internal Medicine, Università degli Studi di Genova, 16132 Genova, Italy; 20 Department of Integrated Surgical and Diagnostic Sciences, Università degli Studi di Genova, 16132 Genova, Italy; 21 IRCCS Ospedale Policlinico San Martino, Unit of Medical Oncology 1, 16132 Genova, Italy; stefania.sciallero@hsanmartino.it; 22 IRCCS Ospedale Policlinico San Martino, Medical Oncology 2, 16132 Genova, Italy; fedececchi@hotmail.it (F.C.); enrica.tanda@gmail.com (E.T.T.); francesco.spagnolo85@gmail.com (F.S.); Paola.Queirolo@ieo.it (P.Q.); 23 Italian Melanoma Intergroup (IMI), 16121 Genova, Italy; segreteria.melanomaimi@gmail.com; 24 Clinical Genetics Branch, Division of Cancer Epidemiology and Genetics, National Cancer Institute, Rockville, MD 20892, USA; goldstea@mail.nih.gov

**Keywords:** familial melanoma, genetic susceptibility, gene panel sequencing, high-penetrance genes, variant interpretation, missing heritability, *CDKN2A*, *ATM*, *BAP1*, *POT1*

## Abstract

The contribution of recently established or candidate susceptibility genes to melanoma missing heritability has yet to be determined. Multigene panel testing could increase diagnostic yield and better define the role of candidate genes. We characterized 273 *CDKN2A/ARF* and *CDK4-*negative probands through a custom-designed targeted gene panel that included *CDKN2A/ARF*, *CDK4*, *ACD*, *BAP1*, *MITF*, *POT1*, *TERF2IP*, *ATM*, and *PALB2*. Co-segregation, loss of heterozygosity (LOH)/protein expression analysis, and splicing characterization were performed to improve variant classification. We identified 16 (5.9%) pathogenic and likely pathogenic variants in established high/medium penetrance cutaneous melanoma susceptibility genes (*BAP1*, *POT1*, *ACD*, *MITF*, and *TERF2IP*), including two novel variants in *BAP1* and 4 in *POT1*. We also found four deleterious and five likely deleterious variants in *ATM* (3.3%). Thus, including potentially deleterious variants in *ATM* increased the diagnostic yield to about 9%. Inclusion of rare variants of uncertain significance would increase the overall detection yield to 14%. At least 10% of melanoma missing heritability may be explained through panel testing in our population. To our knowledge, this is the highest frequency of putative *ATM* deleterious variants reported in melanoma families, suggesting a possible role in melanoma susceptibility, which needs further investigation.

## 1. Introduction

Phenotypic and environmental factors, such as skin phototype, presence of atypical nevi, exposure to sunlight, and sunburn, are known to increase the risk of cutaneous melanoma (CM). However, 5% to 12% of CM cases have a positive family history for this malignancy. Until a few years ago, *CDKN2A/ARF* (cyclin dependent kinase inhibitor 2A) and its binding partner *CDK4* (cyclin dependent kinase 4) were the only known melanoma-predisposition genes tested in clinical practice. Germline pathogenic variants in *CDKN2A/ARF* have been found in 20–45% of familial CM cases [1] and in 11–19% of multiple primary melanomas (MPM) [2] from the Italian population, characterized by a high prevalence of *CDKN2A* pathogenetic variants [3] often associated with pancreatic cancer (PC) and other cancers [4,5]. Conversely, a small number of families carrying pathogenic variants in *CDK4* have been described worldwide, including some Italian families [6,7].

As cancer predisposition syndromes and their associated genes are being clarified, previously unappreciated overlaps are being identified. Indeed, some CM genes are associated with different cancer types, and genes with known predisposition to other cancers may also increase the risk of CM. Several distinct CM syndromes have been defined and genetic tests are now available for the associated causative genes [8].

Recently, novel rare high-risk variants have been identified in *BAP1* (BRCA1 associated protein 1), *POT1* (protection of telomeres 1), *ACD* (adrenocortical dysplasia), *TERF2IP* (telomeric repeat-binding factor-2 interacting protein), and the *TERT* (telomerase reverse transcriptase) promoter primarily by whole exome sequencing (WES) studies, although differences in terms of prevalence have been reported across studies [1,9,10,11,12,13]. *TERT* promoter variants have been reported in just two families [14,15]. Moreover, *MITF* (melanocyte inducing transcription factor) has emerged as an intermediate penetrance risk gene for CM. Namely, carriers of the *MITF* p.Glu318Lys variant have a greater than fivefold increased risk of developing melanoma, especially MPM, with specific dermatological and dermoscopic features and with renal cell carcinoma (RCC) or both compared with non-carriers [16,17,18,19,20,21]. On the basis of this evidence, it has been proposed that the aforementioned genes should be added to *CDKN2A* and *CDK4* in gene panels for routine diagnostic testing [8,22].

Familial clustering of additional cancer types, such as *CDKN2A*-associated PC and p14arf-associated nervous system tumors, has long been described in CM families [23,24,25,26]. Further, in families with pathogenic variants in novel susceptibility genes, CM is part of a broader multi-cancer syndrome [8,27]. In addition to CM, the tumor spectrum of *BAP1* pathogenic variants includes uveal melanoma (UM), mesothelioma, basal cell carcinoma (BCC), renal cell carcinoma (RCC), and BAP1-inactivated melanocytic tumors (BIMTs), which together constitute the *BAP1*-tumor predisposition syndrome (*BAP1*-TPDS) [27]. Moreover, *POT1* pathogenic variants predispose to glioma, thyroid cancer, cardiac angiosarcoma, and colorectal cancer [28,29,30,31]. Lastly, *ACD* and *TERF2IP*, as well as *TERT* promoter variants, are associated not only with early onset and multiple CM, but also with other cancers, although these latter associations are not yet conclusively defined [12].

Pathogenic variants in the above-mentioned genes appear to be extremely rare in the context of non-syndromic familial aggregation (i.e., a familial clustering of only one type of cancer, such as CM). Conversely, recent exome sequencing studies on PC-only families identified pathogenic variants in the *ATM* (ataxia-telangiectasia mutated) [32,33] and *PALB2* (partner and localizer of *BRCA2*) genes [34,35].

*ATM* is a known intermediate penetrance cancer predisposition gene, as heterozygous carriers of *ATM* germline pathogenic variants are at increased risk of developing several types of malignancies [36], including breast cancer (BC) and PC. The findings of genome-wide association studies (GWASs) suggest that *ATM* may be a low-risk melanoma susceptibility locus [37,38], but functional alleles have not yet been identified. In a recent WES study on PC cases from *CDKN2A*-positive and negative CM families, *ATM* loss-of-function (LOF) variants were mostly observed in *CDKN2A*-negative PC patients [39]. In another WES study, a potentially deleterious missense variant in *ATM* detected in a CM family co-segregated with CM in three affected members whose mother developed PC [26].

Most *ATM* variants are, however, of uncertain significance, and the role of *ATM* in CM predisposition has yet to be clarified.

In addition, low penetrance genes such as *MC1R* (melanocortin 1 receptor) and far more than 20 other low penetrance risk loci have recently been associated with CM through Sanger sequencing (SS) and WES studies, as well as GWAS [37,40].

Although the individual alteration of each of these loci is likely not sufficient to drive oncogenesis, the interplay of multiple low penetrance risk alleles may increase personal CM risk above a critical threshold, leading to familial clustering of CM. The validation of a polygenic score is currently underway to verify this hypothesis [41,42,43].

All these issues should be addressed during CM genetic risk assessment in both research and clinical settings, to improve the efficiency of genetic referral, counselling, and testing.

For this study, we performed germline sequencing through a multigene panel that included established and candidate predisposition genes, in a cohort of Italian CM patients deemed at high-risk according to recent established criteria [8], but negative for *CDKN2A/ARF* and *CDK4* pathogenic variants. Our aims as follows: to validate, in CM cases with a high risk of genetic susceptibility, a comprehensive gene panel containing all established CM susceptibility genes identified so far, and a few novel candidates; to assess the frequency of pathogenic variants; and to evaluate the potential impact of this panel in the clinical practice in terms of increased diagnostic yield and of interpretational challenges of novel variants. Under the hypothesis that part of the aggregation of PC seen in our CM families could be ascribed to variants in *ATM* and *PALB2*, we decided to include these genes in our panel, with the purpose of investigating their potential role in CM predisposition.

## 2. Results

### 2.1. Panel Validation

To determine the sensitivity and specificity of our next-generation sequencing (NGS) platform and bioinformatics algorithm, we first sequenced 10 DNA samples with known *CDKN2A/ARF*, *CDK4*, and *MC1R* germline variants previously found through SS. The NGS results were fully concordant with the previous SS results. All samples from the patients included in the study, previously sequenced for *CDKN2A/ARF* and *CDK4*, were confirmed to be negative by NGS. A 100% concordance in single-nucleotide polymorphisms (SNPs) detection in *CDKN2A/ARF* and *MC1R* was observed.

### 2.2. Detection Yield and Variant Interpretation in Established and Candidate Melanoma Predisposition Genes

Out of 273 probands who underwent gene panel testing, we identified 16 (5.9%) pathogenetic or likely pathogenic variants in established high/intermediate penetrance CM susceptibility genes such as *BAP1* (2.2%; *n* = 6), *POT1* (0.7%; *n* = 2), *ACD* (0.37%; *n* = 1), and *MITF* (2.6%; *n* = 7). We also found eight variants of uncertain significance (VUS): one in *BAP1*, six in *POT1*, and one in *TERF2IP*.

In addition, we found four deleterious variants and five potentially deleterious variants (3.3%) as well as six rare VUS in *ATM*, whereas no rare variants were found in *PALB2*. The detection yield of each gene, considering deleterious and potentially deleterious variants, with or without VUS, respectively, is reported in the titles of the following gene-specific paragraphs (for pedigrees, see Figure S1).

#### 2.2.1. BAP1 (2.2–2.5%)

Overall, we identified six variants, five frameshift and one non-sense, which were classified as pathogenic, as well as one VUS in the *BAP1* gene. Of these, two frameshift variants were novel, whereas we had already reported the remaining variants in the meta-analysis by Walpole et al. [44], but none of them were found in our control cohort. All families with LOF variants showed the typical features of the *BAP1*-TPDS (OMIM 614327) as well as multiple cases of CM (Table 1).

The patient with the novel c.1337delA variant developed a spitzoid CM at the age of 41, as well as a further CM and two dysplastic nevi—one classified as spitzoid—at the age of 43. Her father had died of UM at the age of 51.

The second novel variant, c.1777dupC, which is predicted to cause a truncated protein after 50 amino acids, was found in a young patient diagnosed with an atypical Spitz nevus/tumor with loss of BAP1 expression and with a second-degree relative deceased owing to RCC at the age of 46.

The patient with c.799_800delCA developed CM and had a brother previously diagnosed with three CMs and a mesothelioma. One year after *BAP1* genotyping, mutation-specific follow-up allowed an early diagnosis of mesothelioma in the proband. We tried to assess loss of heterozygosity (LOH) by tumor DNA sequencing in both melanomas and mesothelioma tissue; unfortunately, low DNA quality limited our analysis, but immunohistochemistry (IHC) showed loss of BAP1 nuclear expression in the mesothelioma sample (see Figure S2).

Additionally, in a patient diagnosed with CM at the age of 52, we identified a missense *BAP1* variant, c.1507T>C p.(Phe503Leu), which was found in The Genome Aggregation Database (gnomAD) with a very low frequency in the non-Finnish European population (f = 3.99e-6).

The patient’s sister developed CM at the age of 23, but segregation in the family could not be verified as *BAP1* germline status was only available for the proband, the family history was not suggestive for a *BAP1* mutation, and the variant was thus classified as VUS.

#### 2.2.2. POT1 (0.7–2.9%)

Multiple variants in the *POT1* gene (four of them novel) were identified, including two pathogenic splicing variants and six missense VUS.

**Figure 1:**
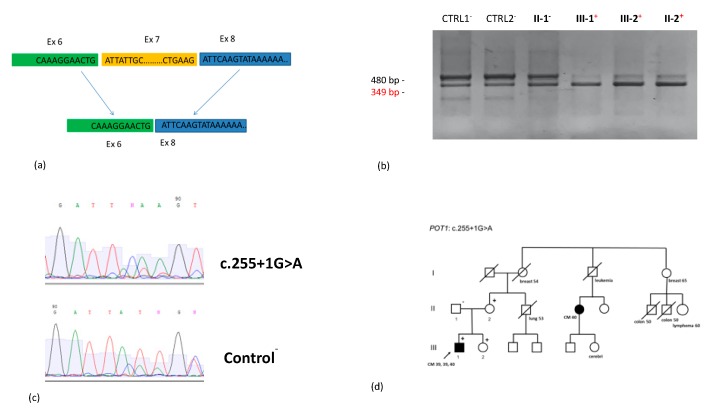
Pedigree with the *POT1* c.255+1G>A variant and the resulting splicing of exon 7. (**a**) Schematic representation of the splicing of exon 7 (113bp) owing to the c.255+1G>A variant. (**b**) Electrophoresis of the cDNA from the four family members, including three (the affected proband, sister, and mother) who carry the variant c.255+1G>A; the father from the unaffected branch of the family who does not carry the variant; and two healthy controls (CTRL-), showing two different transcripts, the shortest resulting from the skipping of exon 7, and overrepresented in carriers compared with non-carriers. (**c**) cDNA sequencing confirmed that the mutant allele produced the shorter isoform, with skipping of exon 7, in a higher proportion of the transcript in carriers vs. non-carriers. The blue arrow indicates the lower relative abundance of the spliced isoform (ex 6–8) in noncarriers vs. carriers (red arrow). (**d**) Pedigree diagram of the family carrying the c.255+1G>A variant. Dark symbol: cutaneous melanoma (CM). Cancer type and age at diagnosis are indicated under each symbol. The proband is indicated by an arrow. +: carrier, −: non-carrier.

The c.255+1G>A novel variant, involving the same canonical splice site of a variant functionally described by Potrony et al. [45], was found in a patient who developed three CMs and several dysplastic nevi, as well as in his unaffected mother and sister. cDNA analysis confirmed the resulting previously described exon 7 skipping, which was overrepresented in carriers compared with non-carriers (approximately 50% vs. 10%) (Figure 1).

The c.1687-1G>A variant, which was predicted to be pathogenic by the MaxEntScan algorithm, and was previously shown to affect transcript splicing by RT-PCR and sequencing by Robles-Espinoza et al. [10], was found in a family with two CM cases and an RCC case.

Three of the six *POT1* missense variants (c.158C>T, c.280C>A, and c.314C>T) were located in the DNA binding domain (OB1). c.158C>T p.(Thr53Ile), predicted to be pathogenic by in silico analysis, was detected in two different CM families: one included individuals with glioblastoma and PC, while the second was a CM-only family, with two affected CM members. In both families, the variant co-segregated with CM. The c.280C>A p.(Gln94Lys) variant, which, according to A.J. Ramsay et al. [46], may disrupt electrostatic interactions with telomeric DNA, was identified in a patient diagnosed with CM at the age of 45, ovarian neoplasm at the age of 23 and papillary peritoneal mesothelioma at the age of 50. The third variant, c.314C>T p.(Thr105Met), is phylogenetically highly conserved (suggesting that this threonine has an essential role in protein function) and was found in a MPM proband who also developed a dysplastic nevus and whose nephew had an atypical Spitz lesion.

Of the remaining missense variants, c.809G>A p.(Ser270Asn) was found in a patient with more than 50 melanocytic nevi who developed nine CMs and was diagnosed with multiple myeloma at the age of 54. After bone marrow transplantation, the patient developed an oligodendroglioma and a mixosarcoma. Moreover, his mother developed a spinal cord tumor at the age of 65.

The last missense variant, c.1400C> p.(Ser467Leu), was found in a patient who developed two CMs and whose mother developed a CM at the age of 50 and a glioblastoma at the age of 79.

#### 2.2.3. ACD (0.36%), TERF2IP (0.36%), and TERT (0%)

We found the c.866_867delCT frameshift variant in *ACD* that leads to a premature truncating protein in a patient affected by multiple CM and with a family history of CM and a relative who died of PC at the age of 55. Additionally, an MPM patient, with no family history of CM, had a VUS in the *TERF2IP* gene, c.258C>G p.(Asp86Glu).

None of the 273 probands carried the c.-57T>G or other germline pathogenic *TERT* promoter variants.

#### 2.2.4. MITF (2.6%)

The *MITF* c.952G>A p.(Glu318Lys) risk variant was detected in 7 out 273 probands, four of whom had a history of MPMs and dysplastic nevi, one was also affected by RCC, and another who had a second degree relative also diagnosed with CM.

#### 2.2.5. PC-Associated Candidate Genes: ATM (3.3–5.5%) and PALB2

Nine potentially pathogenic (four deleterious and five potentially deleterious variants) and six VUS were found in *ATM*. c.3275C>A p.(Ser1092Ter) was detected in a CM proband with a second degree relative affected by PC. Three frameshift variants, predicted to cause truncated proteins owing to premature stop codons, were detected in families with MPM, UM, and other cancers (Table 2).

Of these, c.4451delT was found in a proband who was treated 10 years earlier with cyclosporine and radiotherapy for a Hodgkin’s lymphoma, and subsequently developed three CMs, two at the age of 45 and one at age 46. The proband also reported a cousin diagnosed with CM who died at the age of 33, while his mother developed a colon adenocarcinoma at age 71 and PC at age 74. A cousin of the mother developed PC at the age of 76. In consideration of personal and family history, the presence of the *ATM* variant was reported to the proband in a research context, with emphasis on the uncertainty about the role of *ATM* in melanoma and PC development and the absence of a standardized surveillance protocol. As the patient was under active follow-up for his prior diseases, a specific PC surveillance was not recommended. Recently, almost three years later, the routine follow-up allowed diagnosis of PC for this patient. The diagnosis was confounded by an acute pancreatitis and uncertainty at computed tomography (CT) and positron emission tomography (PET) scans followed by cholangio-nuclear magnetic risonance (NMR). Shortly after histologic diagnosis was confirmed at ultrasound (US) endoscopy, the disease rapidly evolved with hepatic metastastases.

c.5979_5983delTAAAG, which causes a premature stop codon after 23 aa, was found in a proband who developed two Ums at the age of 41 and a CM 10 years later.

The last frameshift variant, c.8319_8323dupTGTCC, was found in a woman who developed two CMs at the ages of 48 and one at 53, as well one BCC and a BC (at the ages of 49 and 53, respectively).

The c.3576G>A p.(Ser1135_Lys1195del58) variant, a splicing mutation resulting in the skipping of the entire exon 24 of the *ATM* gene [47], was found in three probands belonging to CM families, two of whom developed MPM and have a first degree relative affected by CM.

The c.5750G>C p.(Arg1917Thr) missense variant was found in two probands. The first one, a CM patient diagnosed at the age of 47, belonged to a family in which the variant co-segregated with the sister (CM at the age of 43) and their father developed prostate, bladder, colon cancer, and UM (at the ages of 70, 71, 72, and 74, respectively).

The second proband harboring the p.(Arg1917Thr) variant was an apparently sporadic MPM patient, with no other tumor in the family. This patient was also a carrier of a *MITF* p.(Glu318Lys) variant.

More details on family histories are available in Figure S1.

In addition to the above-mentioned variants, we found six additional missense variants, all of uncertain significance (Table 2). Conversely, no rare variants were found in the *PALB2* gene.

#### 2.2.6. CDKN2A/ARF and CDK4

We did not find previously undetected pathogenic variants in *CDK4* and *CDKN2A/ARF*, although we included regulatory regions, for example, the whole *CDKN2A/ARF* 5′UTR, as we recently showed that nearly half of the rare 5′UTR germline variants are potentially pathogenic, owing to their impact on *CDKN2A/ARF* internal ribosome entry site (IRES)-mediated translation [48,49].

## 3. Discussion

The overall contribution of recently identified CM predisposition genes (*ACD*, *BAP1*, *POT1*, *MITF*, and *TERF2IP*) to melanoma’s missing heritability is estimated to be about 2% [22]. Initial findings of pathogenic variants in *POT1*, however, considered the second most frequently mutated high penetrance gene after *CDKN2A/ARF*, showed a higher frequency, probably owing to the presence of founders in the studied populations [8,10]. Therefore, these reports need to be confirmed by further studies. Currently, pathogenic variants in recently identified CM predisposition genes are detected at a frequency similar to that of pathogenic *CDK4* variants, which was routinely screened in familial and MPM patients together with *CDKN2A/ARF*. These data supported the inclusion of these genes in clinical genetic testing panels [8,22,50,51]. With this study, we evaluated the efficiency and the potential clinical benefit of a customized multi-gene panel for CM risk that could enhance genetic testing offered to Italian CM families and MPM patients. In addition to the whole *CDKN2A/ARF* gene (including the 5′UTR), and exon 2 of *CDK4*, this panel included the novel established high penetrance genes, that is, *ACD*, *BAP1*, *POT1*, *TERF2IP*, as well as the intermediate penetrance gene *MITF*. *TERT* promoter was analyzed by SS. We also included *ATM* and *PALB2* for research purposes, excluding them from the clinical report, under the assumption that their variants could play a role in families with known cancer associations, that is, CM and PC [26,39,52,53].

Overall, the detection yield of pathogenic/likely pathogenic variants in established melanoma risk genes was 5.86%. When deleterious and potentially deleterious *ATM* variants were also included, the detection yield rose to 9.16%, the highest estimate described so far to our knowledge.

However, the association between variants in *ATM*, mostly known for being associated with a low-intermediate risk of other cancers, and CM risk is not yet clearly established.

Indeed, our study may provide useful information to help tackle those issues hampering the implementation of research discoveries in clinical practice. An increase in the detection yield needs to be integrated with a thorough evaluation of the pathogenicity of variants, especially novel ones, because of the potential clinical impact. Any VUS is subject to future reassessments of clinical impact. Further, the detection of a pathogenic variant in a gene such as *BAP1*, which is related to a complex syndrome that includes several potentially lethal cancers, should be reported to aid clinical management, even though the complete tumor spectrum and related tumor-specific penetrance and surveillance recommendations are not definitively drawn.

In our cohort, the overall detection rate of LOF in *BAP1* was 2%. However, if we restricted assessment to patients/families meeting criteria [8] for the *BAP1* syndrome (*n* = 36), our detection rate rose to 16.7%. For an appropriate clinical management of BAP1-TPDS patients, we suggested a surveillance plan based on preliminary proposals [27], informing the patients about the risk of additional cancers associated with the syndrome and about the uncertainty of the efficacy of surveillance for some of these cancers.

For patients with *MITF* p.(Glu318Lys), based on the association of a higher risk of MPM, dysplastic nevi, and RCC, and on the basis of our data [18], we propose the inclusion of this gene in clinical diagnostic testing and suggest that an annual abdominal ultrasound should be integrated with the dermatological surveillance for the carriers, even though tailored surveillance for cancers other than CM is not standardized [51].

Specific recommendations are not yet available for multiple variants/genes such as *POT1* variants. Therefore, the impact on surveillance of these novel melanoma risk genes should be considered and communicated, bearing in mind that they will not be definitive until a consensus is reached based on the results of international multicentric studies, such as those conducted by the BIG consortium (BAP1 interest group, http://www.bap1.org) for the *BAP1* gene.

Interestingly, our study cohort showed a higher rate of ATM variants than that reported in recent WES and WGS studies that included melanoma [54,55]. One reason could be that those studies were performed on melanoma patients, regardless of family history, whereas our cohort included high-risk individuals, more likely to carry germline risk alleles. However, these data are not enough to assess whether ATM is involved in melanoma predisposition.

The finding of *ATM* LOF variants in our study cohort is in line with previous findings on the association between *ATM* and CM/PC risk [26,39]. In fact, four deleterious and five potentially deleterious variants were found, three of them in melanoma-prone families that also included PC cases. Co-segregation in PC cases, however, could not be determined. Therefore, PC occurrence in these families may only partially explain this high detection rate, although one *ATM* positive patient developed both melanoma and PC. Therefore, further studies are needed to determine whether *ATM* is a risk factor for melanoma.

Moreover, surveillance for other cancers for which *ATM* is a medium penetrance predisposition gene (for instance, BC) is still under debate and discussion [53,56,57,58].

Overall, the search for variants in recently identified genes consistently associated with risk of CM is a step forward to improve diagnostic genetic-testing panels and to evaluate differences in geographical distribution. Recently, Potjer et al. [59] found no variants in *POT1*, and occurrence of UM and mesothelioma in three *BAP1* positive families in a large multigene panel study of 451 Dutch families with an overall diagnostic yield of 4%, principally explained by the identification of 15 *MITF* positive families (3.3%). Casula et al., in a cohort of Italian MPM patients, used a panel containing the same genes we tested (with the exception of *MITF*), and found a lower pathogenic variant rate, considering the American College of Medical Genetics on Genomics (ACMG) variant classification (3%) [60]. *CDKN2A* pathogenic variants were also less frequent (3.84%) [2,3]. More work is needed to evaluate different diagnostic panels, within and across geographic areas.

This study naturally has some limitations. We were limited in our ability to verify co-segregation in most families because affected family members were deceased or not available, resulting in some variants being classified as VUS. Unfortunately, this is a common issue in the diagnostic setting. In addition, we recognize the importance of functional analysis and agree that segregation analysis coupled with functional assays is currently the best approach to classify gene variants. Functional validation will be part of future studies that will help define the role of these variants/genes in melanoma susceptibility. Another minor limitation is that the panel did not include several rare/very rare recently proposed candidate melanoma genes such as GOLM1 (golgi membrane protein 1) [61], POLE (DNA polymerase epsilon, [62]), NEK11 [63], EBF3, OCA2, TYR, TYRP1, and SLC45A2 [26,59,64,65,66], reviewed in [67].

## 4. Materials and Methods

### 4.1. Study Cohort

We selected 273 index cases, consecutively enrolled at our center for genetic testing and tested negative for CDKN2A and CDK4. The genetic testing assessment is based on a clinical score that incorporates criteria previously proposed for genetic testing for CM susceptibility [8], accounting for the differences among areas with high or low incidence. It applies criteria to proceed with genetic testing for cancers other than melanoma in families with members affected by CM: 167 probands belonged to families with two or more affected CM members, 84 were apparently sporadic MPM patients, and 22 were apparently non-familial CMM or atypical Spitz nevus cases whose first/second degree relatives were diagnosed with BAP1-TPDS related cancer or PC. When a pathogenic, likely pathogenic, or VUS variant was identified, co-segregation analysis was performed, extending the analysis to other affected family members, when available, for a total of 293 individuals tested. All patients were interviewed by a medical geneticist in the context of clinical genetic counselling, using a standardized questionnaire that comprised both family history and environmental exposures. In addition, 80 cancer-free controls from the general population were selected, tested, and administered the same questionnaire. All subjects enrolled in this study signed an informed consent for genetic testing and genetic-based research under local Institutional Review Board (IRB) approved protocols (CE AOU San Martino Genova 10/2010).

### 4.2. Panel Design

We performed custom targeted sequencing covering coding exons and splice junctions of *CDKN2A/ARF* (including exon 1 Beta and 5′UTR and promoter of exon 1alpha upstream to 400 bp from ATG) [68], *CDK4 exon2*, *ACD*, *BAP1*, *MITF exon 10*, *POT1*, *TERF2IP*, *ATM*, and *PALB2*. Our panel covered 98% of the regions targeted. The remaining 2% of uncovered regions were analyzed through direct sequencing. *TERT* promoter sequence was excluded from the panel design owing to low coverage and was instead analyzed through direct sequencing for all cases. The panel contained 198 amplicons for a total size of 55.5 Kb.

### 4.3. Targeted Sequencing and Variant Selection and Interpretation

In brief, libraries were prepared using the Ion Chef System, and then pooled and loaded into PGM or S5 next-generation sequencing systems (Thermo Fisher Scientific, Waltham, MA, USA) for automated sequencing. Ion sequencing data were analyzed using the Torrent Variant Caller software within the Torrent Suite. Annotation was performed by uploading BAM files into Ion Reporter 5.10 and wAnnovar [69], and both tools were used to annotate genetic variants. Only single nucleotide variants and small indels covered for more than 20 reads were filtered in. Sequencing data were filtered by frequency, in order to isolate clinically relevant mutations only in high/medium risk genes. Variants were classified as pathogenic, likely pathogenic, VUS, likely benign, or benign based on the published ACMG recommendations [70], for known melanoma genes. For *ATM*, however, we used the term deleterious because the gene is not considered an established melanoma predisposition gene, or a gene definitively known to cause the disease. Variants were named according to the Human Genome Variation Society nomenclature (HGVS, https://varnomen.hgvs.org/). All identified putative pathogenic variants and VUS were validated by SS.

### 4.4. Next-Generation Sequencing (NGS) Analysis

We filtered out common variants based on their frequency (MAF higher than 0.01) in the gnomAD (Genome Aggregation Database https://gnomad.broadinstitute.org/) public database or in our cohort of 80 healthy controls sequenced with the same panel. An additional filtering of non-synonymous variants was performed according to the following tools: SIFT (sorting intolerant form tolerant, Polyphen 2, LRT (likelihood ratio test), mutation taster, CADD (combined annotation-dependent depletion) score > 15, and ClinVar. Synonymous variants were analyzed using Human Splicing Finder (http://www.umd.be/HSF3/) and those without a predicted effect on splicing were excluded. Frameshift, splicing, and stop-gain variants were defined as LOF.

### 4.5. BAP1 LOH/Protein Expression by Immunohistochemistry (IHC)

BAP1 LOH/protein expression by IHC was performed to verify BAP1 loss/inactivation using a mouse monoclonal antibody raised against amino acids 430–729 of human BAP1 (C-4, Cat. No. sc-28383; Santa Cruz, CA, USA) on Formalin-Fixed, Paraffin-Embedded (FFPE) mesothelioma sections from patients included in the study cohort.

### 4.6. Splicing Analysis

To verify whether the variant c.255+1G>A alters splicing in *POT1*, as described in Potrony et al. [45], in which they tested the variant c.255G>A, we isolated total RNA from lymphoblastoid cells with TRIzol (Invitrogen, Carlsbad, CA, USA) and cDNA synthesis was performed with the High-Capacity cDNA Reverse Transcription Kit (Applied Biosystem, Foster City, CA, USA) using random primers. PCR primers were designed to cover exons 6–9 of the *POT1* gene and the same primers were used for direct sequencing. The PCR products of the subjects carrying the variant and different controls were loaded on an agarose gel. *POT1* NM_015450 was used as a reference sequence.

## 5. Conclusions

Multigene panel sequencing, steadily becoming more affordable and accessible, has improved cancer risk assessment in the clinical practice by allowing a more accurate identification and surveillance of families at risk of CM. However, insights on the definition of novel cancer clusterings, or a complex polygenic interplay that influences the clinical expression of a pathogenic variant, require larger case-control studies to allow a finer personalization of cancer risk estimates. To the best of our knowledge, this is the first study to report a high percentage of deleterious *ATM* variants in melanoma families (3.3%). In fact, the finding of *ATM* deleterious or potentially deleterious variants in nearly 3.3% of our families, and an additional 2.2% of rare VUS, has led to a multicenter international collaboration, currently ongoing, to define the role of *ATM* as a susceptibility gene to CM. The fact that the risk for some other types of cancer is not completely defined in CM families represents one of the challenges arising from wide-range genetic testing, as well as a novel opportunity to identify potentially lethal cancer-prone syndromes.

## Figures and Tables

**Table 1:** Rare variants in melanoma high penetrance genes *ACD*, *BAP1*, *POT1*, and *TERF2IP* and in medium penetrance gene *MITF*.

Case Index (Age of Diagnosis)	Gene ^1^	Description and Consequence	Type	ACMG Classification ^2^	DbSNP ^3^	gnomAD ^4^	CADD ^5^	SIFT ^6^	Polyphen ^7^	LRT ^8^	Mutation Taster	Other Cancer in Family	Clin Var ^9^	Co-Segregation ^10^	Total No. of cm/um Cases in Family
MPM (58)	*ACD*	c.866_867delCT; p.(Pro289ArgfsTer28)	frameshift	LP	rs777476880	2.44e-05	-	-	-	-	-	BC, LC, PC	-	ND	3 CM
CM (31)	*BAP1*	c.327_328insGA; p.(Pro110AspfsTer4)	frameshift	P	-	-	-	-	-	-	-	2 MES	-	ND	1 CM
UM (45), RCC (40)	*BAP1*	c.639dupT; p.(lIe214TyrfsTer29)	frameshift	P	-	-	-	-	-	-	-	2 KC, 2 UM		Y	3 UM
CM (53), MES (55)	*BAP1*	c.799_800delCA; p.(Gln267AlafsTer16)	frameshift	P	-	-	-	-	-	-	-	3 CM, LC, 2 MES		Y	4 cCM
MPM (41, 43)	*BAP1*	c.1337delA; p.(Asn446ThrfsTer125)	frameshift	P	-	-	-	-	-	-	-	UM, BC		ND	1 UM, 3 CM
CM (52)	*BAP1*	c.1507T>C; p.(Phe503Leu)	missense	VUS	rs745959970	3.99e-06	23.6	D	D	D	D	CM		ND	2 CM
AST (23)	*BAP1*	c.1777dupC; p.(Gln593ProfsTer50)	frameshift	P	-		-	-	-	-	-	RCC	-	ND	1 AST
AST (35), AGM	*BAP1*	c.1939G>T; p.(Glu647Ter)	non-sense	P	-		-	-	-	-	-	BC, BCC, UM		Y	1 AST, 1 UM
CM (26)	*POT1*	c.158C>T;p.(Thr53Ile)	missense	VUS	-	-	21.3	D	D	D	D	3 CM, GLB, PC, HL	-	Y	4 CM, 1 UM
MPM (66, 66)	*POT1*	c.158C>T; p.(Thr53Ile)	missense	VUS	-	-	21.3	D	D	D	D	CM	-	Y	3 CM
MPM (39, 39, 40)	*POT1*	c.255+1G>A	splicing	P	-	-	-	-	-	-	-	CM, BC, CC, LC, L	-	ND	4 CM
CM (45), OC (23), MES (50)	*POT1*	c.280C>A; p.(Gln94Lys)	missense	VUS	-	-	22.8	D	D	D	D	LC	-	ND	1 CM
MPM (46, 52)	*POT1*	c.314C>T; p.(Thr105Met)	missense	VUS	-	-	28.8	D	D	D	D	AST, BC, LC	-	ND	2 CM
MPM (40), GL, MY	*POT1*	c.809G>A; p.(Ser270Asn)	missense	VUS	rs587777477	-	23.2	D	B	D	D	LC, S	RF	ND	9 CM
MPM (40, 52)	*POT1*	c.1400C>T;p.(Ser467Leu)	missense	VUS	rs1410842025	-	17.52	D	PD	N	D	CM, GLB	-	ND	3 CM
CM (17)	*POT1*	c.1687-1G>A	splicing	P	rs587777473	4.28e-06	-	-	-	-	-	CM, RCC	RF	Y	2 CM
MPM (40, 51)	*TERF2IP*	c.258C>G; (p.Asp86Glu)	missense	VUS	rs752446617	6.14e-06	23.9	T	B	D	D	BLC, BCC	-	ND	2 CM
MPM (32)	*MITF*	c.952G>A; p.Glu318Lys	missense	LP	rs149617956	1.36e-03	27.9	D	P	D	D	-	UNC	ND	10 CM
CM (23)	*MITF*	c.952G>A; p.Glu318Lys	missense	LP	rs149617956	1.36e-03	27.9	D	P	D	D	CM	UNC	Y	2 CM
MPM (59)	*MITF*	c.952G>A; p.Glu318Lys	missense	LP	rs149617956	1.36e-03	27.9	D	P	D	D	KD	UNC	ND	3 CM
CM (39), TC (38)	*MITF*	c.952G>A; p.Glu318Lys	missense	LP	rs149617956	1.36e-03	27.9	D	P	D	D		UNC	ND	1 CM
MPM (62, 62, 72)	*MITF*	c.952G>A; p.Glu318Lys	missense	LP	rs149617956	1.36e-03	27.9	D	P	D	D		UNC	ND	3 CM
MPM (45, 46)	*MITF*	c.952G>A; p.Glu318Lys	missense	LP	rs149617956	1.36e-03	27.9	D	P	D	D	BC	UNC	ND	2 CM
MPM (52, 62, 65)	*MITF*	c.952G>A; p.Glu318Lys	missense	LP	rs149617956	1.36e-03	27.9	D	P	D	D	CM	UNC	ND	4 CM

^1^ Gene reference: ACD LRG_1237 (NM_001082486.2); BAP1: LRG_529 (NM_004656.2); POT1 (NM_015450); TERF2IP: LRG_1084 (NM_018975.3); MITF: LRG_776 (NM_000248.3). ^2^ ACMG: American College of Medical Genetics and Genomics; ^3^ dbSNP: single nucleotide polymorphism database; ^4^ gnomAD: genome aggregation database; ^5^ CADD: combined annotation-dependent depletion; ^6^ SIFT: sorting intolerant from tolerant; ^7^ PolyPhen: polymorphism phenotyping; ^8^ LRT: likelihood ratio test; ^9^ Clin Var (ClinVar aggregates information about genomic variation and its relationship to human health); ^10^ co-segregation analysis of variant with melanoma phenotype in the family (Y: the observed variant co-segregates in at least two affected members; ND: not done). Abbreviations: *ACD*: adrenocortical dysplasia; *BAP1*: BRCA1 associated protein 1; *POT1*: protection of telomeres 1; *TERF2IP*: telomeric repeat-binding factor-2 interacting protein; *MITF:* melanocyte inducing transcription factor; BC: breast cancer; BCC: basal cell carcinoma; BLC: bladder cancer; CC: colon cancer; CM: cutaneous melanoma; D: deleterious; GC: gastric cancer; HL: Hodgkin lymphoma; LX: larinx cancer; LP: likely pathogenic; MES: malignant mesothelioma; MPM: multiple primary melanoma; ON: oncocytoma; P: pathogenic; PC: pancreatic cancer; PR: prostate cancer; T: tolerant; UC: uterine cancer; UM: uveal melanoma; UNC: uncertain; VUS: variant of unknown significance. The variants in bold are novel.

**Table 2:** Rare variants of interest in *ATM* candidate gene.

Case Index (Age of Diagnosis)	Gene ^1^	Description and Consequence	Type	ACMG Classification ^2^	DbSNP ^3^	gnomAD ^4^	CADD ^5^	SIFT ^6^	Polyphen ^7^	LRT ^8^	Mutation Taster	Other Cancer in Family	Clin Var ^9^	Co-Segregation ^10^	Total No. of cm/um Cases in Family
CM (24)	*ATM*	c.1516G>T:p.(Gly506Cys)	missense	VUS	rs587779816		29.0	D	P	D	D	ON, PC	UNC	ND	1 CM
CM (48)	*ATM*	c.1595G>A:p.(Cys532Tyr)	missense	VUS	rs35963548		21.0	D	D	D	D	2 CM, BC	UNC	ND	3 CM
CM (47)	*ATM*	c.3275C>A;p.(Ser1092Ter)	non sense	P	-	-	-	-	-	D	D	PC, LC	-	ND	1 CM
MPM (42, 42)	*ATM*	c.3576G>A;p.(Ser1135_Lys1195del58)	splicing	LP	rs587776551	1.63e-05	-	-	-	-	-	CM	P	ND	3 CM
CM (49)	*ATM*	c.3576G>A;p.(Ser1135_Lys1195del58)	splicing	LP	rs587776551	1.63e-05	-	-	-	-	-	CM, UC	P	ND	2 CM
MPM (40, 40)	*ATM*	c.3576G>A;p.(Ser1135_Lys1195del58)	splicing	LP	rs587776551	1.63e-05	-	-	-	-	-	MPM, BCC, PC, PR	P	Y	4 CM
MPM (35, 63)	*ATM*	c.3934A>G:p.(Arg1312Gly)	missense	VUS	rs864622137		23.3	D	D	D	D	LC, BR, GC	UNC	ND	2 CM
CM (45)	*ATM*	c.4049C>T:p.(Thr1350Met)	missense	VUS	rs587781785		27.0	T	D	D	D	CM	UNC	ND	3 CM
CM (50)	*ATM*	c.4306C>T:p.(His1436Tyr)	missense	VUS	rs544891616		17.09	T	D	D	D	2 KD	UNC	ND	1 CM
HL (35), MPM (45, 45, 46), BCC (49), PC (50)	*ATM*	c.4451delT:p.(Met1484ArgfsTer15)	frameshift	P	-		-	-	-	-	-	CM, CC, LC, PC	-	ND	4 CM
CM (47)	*ATM*	c.5750G>C:p.(Arg1917Thr)	missense	LP	rs377289524	1.22e-05	25.6	T	D	D	D	CM, UM, BC, BLC, PR, CC, GC	UNC	Y	2 CM, 1 UM
MPM (45, 46)	*ATM*	c.5750G>C:p.(Arg1917Thr)	missense	LP	rs377289524	1.22e-05	25.6	T	D	D	D	PR	UNC	ND	2 CM
2 UM (41), CM (51)	*ATM*	c.5979_5983delTAAAG; p.(Ser1993ArgfsTer23)	frameshift	P	rs876660134	8.13e-06	-	-	-	-	-	BC, GC, LX	P	ND	1 CM, 2 UM
MPM (48, 48, 53), BCC (49)	*ATM*	c.8319_8323dupTGTCC; p.(Pro2775LeufsTer33)	frameshift	P	rs1060501552		-	-	-	-	-	MPM, BCC, PC, PR	P	ND	5 CM
CM (43)	*ATM*	c.8557A>G:p.(Thr2853Ala)	missense	VUS	-		27.7	D	D	D	D	MES	UNC	ND	1 CM

^1^ Gene reference: *ATM* LRG_135 (NM_000051.3). ^2^ ACMG: American College of Medical Genetics and Genomics; ^3^ dbSNP: single nucleotide polymorphism database; ^4^ gnomAD: genome aggregation database; ^5^ CADD: combined annotation-dependent depletion; ^6^ SIFT: sorting intolerant from tolerant; ^7^ PolyPhen: polymorphism phenotyping; ^8^ LRT: likelihood ratio test; ^9^ Clin Var (ClinVar aggregates information about genomic variation and its relationship to human health); ^10^ co-segregation analysis of variant with melanoma phenotype in the family (Y: the observed variant co-segregates in at least two affected members; ND: not done). Abbreviations; *ATM*: ataxia-telangiectasia mutated; BC: breast cancer; BCC: basal cell carcinoma; BLC: bladder cancer; CC: colon cancer; CM: cutaneous melanoma; D: deleterious; GC: gastric cancer; HL: Hodgkin lymphoma; LX: larinx cancer; LP: likely pathogenic; MES: malignant mesothelioma; MPM: multiple primary melanoma; ON: pncocytoma; P: pathogenic; PC: pancreatic cancer; PR: prostate cancer; T: tolerant; UC: uterine cancer; UM: uveal melanoma; UNC: uncertain; VUS: variant of unknown significance. The variants in bold are novel.

## Supplementary Materials

The following are available online at https://www.mdpi.com/2072-6694/12/4/1007/s1, Figure S1: Pedigree diagrams from the families carrying loss of function variants in *BAP1*, *POT1*, *ATM*, *ACD* genes; Figure S2: Pedigree diagram from the family with the rare c.799_800delCA *BAP1* germline variant.
