# Supplementary material for: Insights into Genetic Susceptibility to Melanoma by Gene Panel Testing: Potential Pathogenic Variants in ACD, ATM, BAP1, and POT1
Source: Cancers (Basel). 2020 Apr 19;12(4):1007. doi: 10.3390/cancers12041007 (PMC7226507; doi:10.3390/cancers12041007)
Supplement: Supplementary file 1 [file cancers-12-01007-s001.pdf]

cancers

Article

Insights into genetic susceptibility to melanoma by gene panel testing: potential pathogenic variants in *ACD, ATM, BAP1,* and *POT1*

Supplementary Material

Lorenza Pastorino [1,2,†], Virginia Andreotti [1,2,†], Bruna Dalmasso [1,2,†], Irene Vanni [1,2], Giulia Ciccarese [1,2], Mario Mandalà [3], Giuseppe Spadola [4], Maria Antonietta Pizzichetta [5], Giovanni Ponti [6], Maria Grazia Tibiletti [7], Elena Sala [8], Maurizio Genuardi [9,10], Pietro Chiurazzi [9,10], Gabriele Maccanti [11], Siranoush Manoukian [12], Serena Sestini [13], Rita Danesi [14], Valentina Zampiga [15], Roberta La Starza [16], Ignazio Stanganelli [17], Alberto Ballestrero [2,18], Luca Mastracci [2,19], Federica Grillo [2,19], Stefania Sciallero [20], Federica Cecchi [21], Enrica Teresa Tanda [21], Francesco Spagnolo [21], Paola Queirolo [21], Italian Melanoma Intergroup (IMI)[22], Alisa M. Goldstein [23], William Bruno[1,2,†] and Paola Ghiorzo [1,2,†,*]

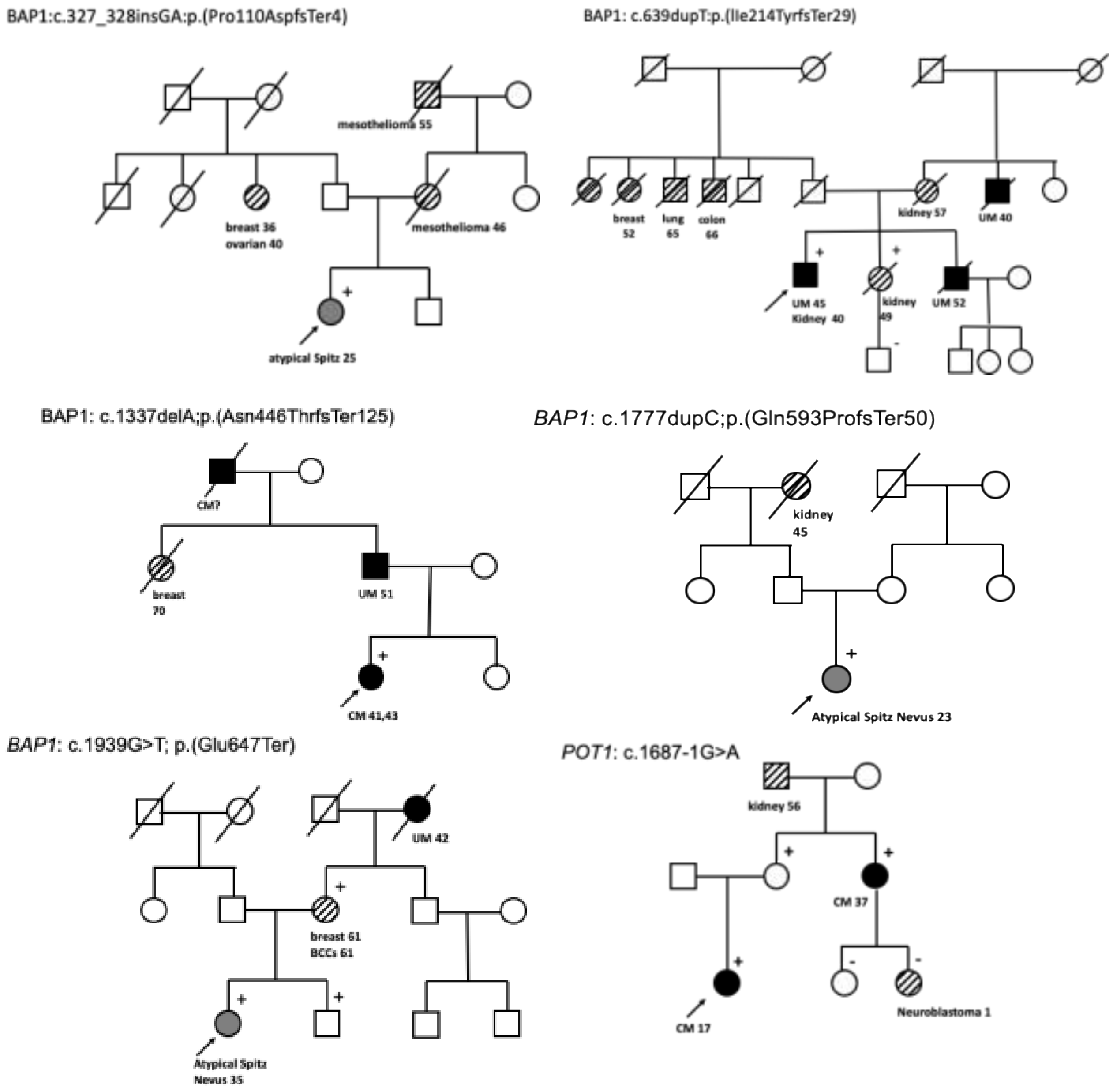
BAP1:c.327_328insGA:p.(Pro110AspfsTer4)
mesothelioma 55
breast 36
ovarian 40
mesothelioma 46
atypical Spitz 25
BAP1: c.639dupT:p.(Ile214TyrfsTer29)
breast 52
lung 65
colon 66
kidney 57
UM 40
UM 45
Kidney 40
kidney 49
UM 52
BAP1: c.1337delA;p.(Asn446ThrfsTer125)
CM?
breast 70
UM 51
CM 41,43
BAP1: c.1777dupC;p.(Gln593ProfsTer50)
kidney 45
Atypical Spitz Nevus 23
BAP1: c.1939G>T; p.(Glu647Ter)
UM 42
breast 61
BCCs 61
Atypical Spitz Nevus 35
POT1: c.1687-1G>A
kidney 56
CM 37
CM 17
Neuroblastoma 1

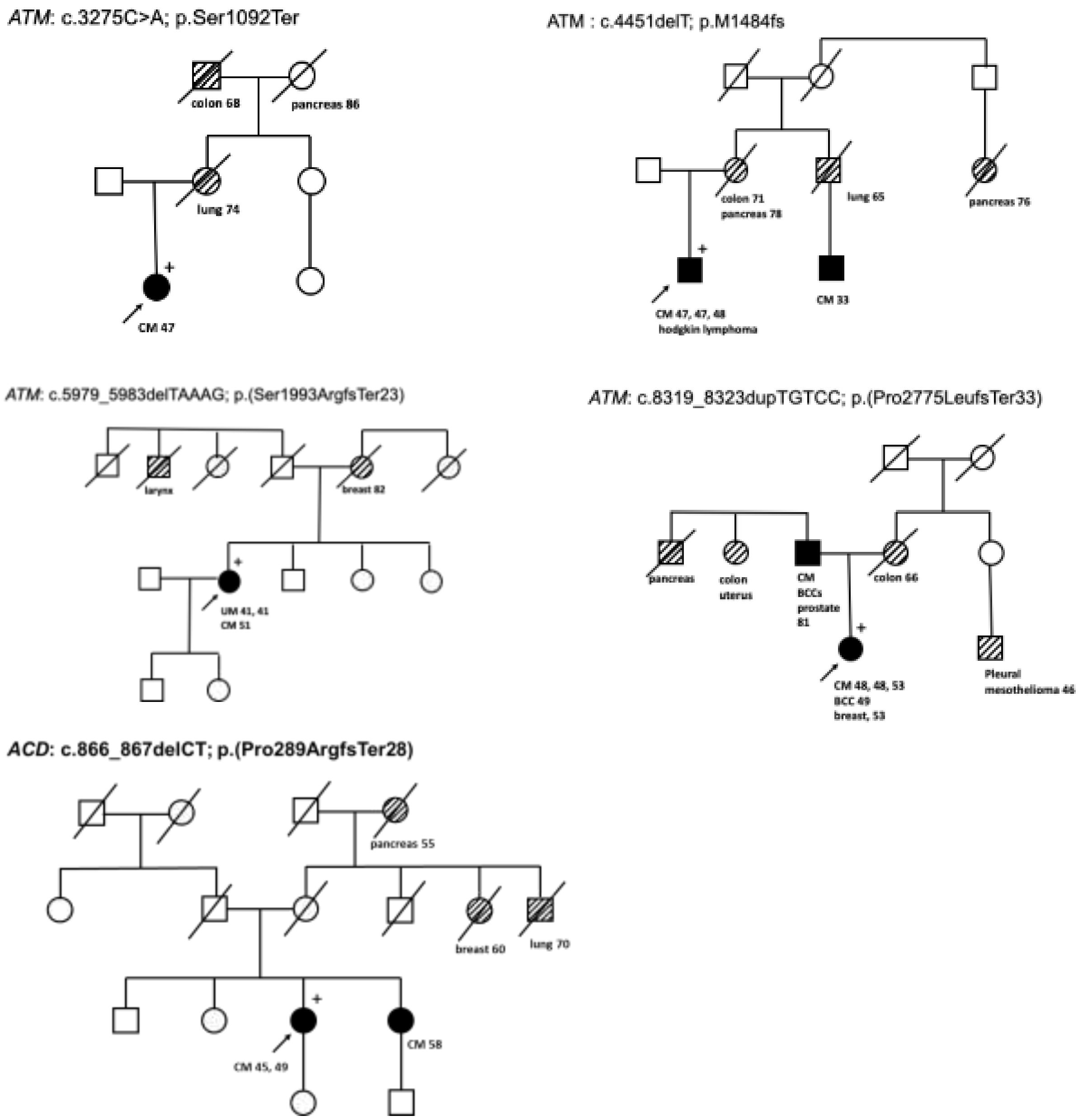

Figure S1. Pedigree diagrams from families carrying loss of function variants in *BAP1, POT1, ATM* and *ACD* genes. Dark symbol: cutaneous melanoma (CM), or uveal melanoma (UM). Cancer type and age at diagnosis is indicated under each symbol. The proband is indicated by an arrow.

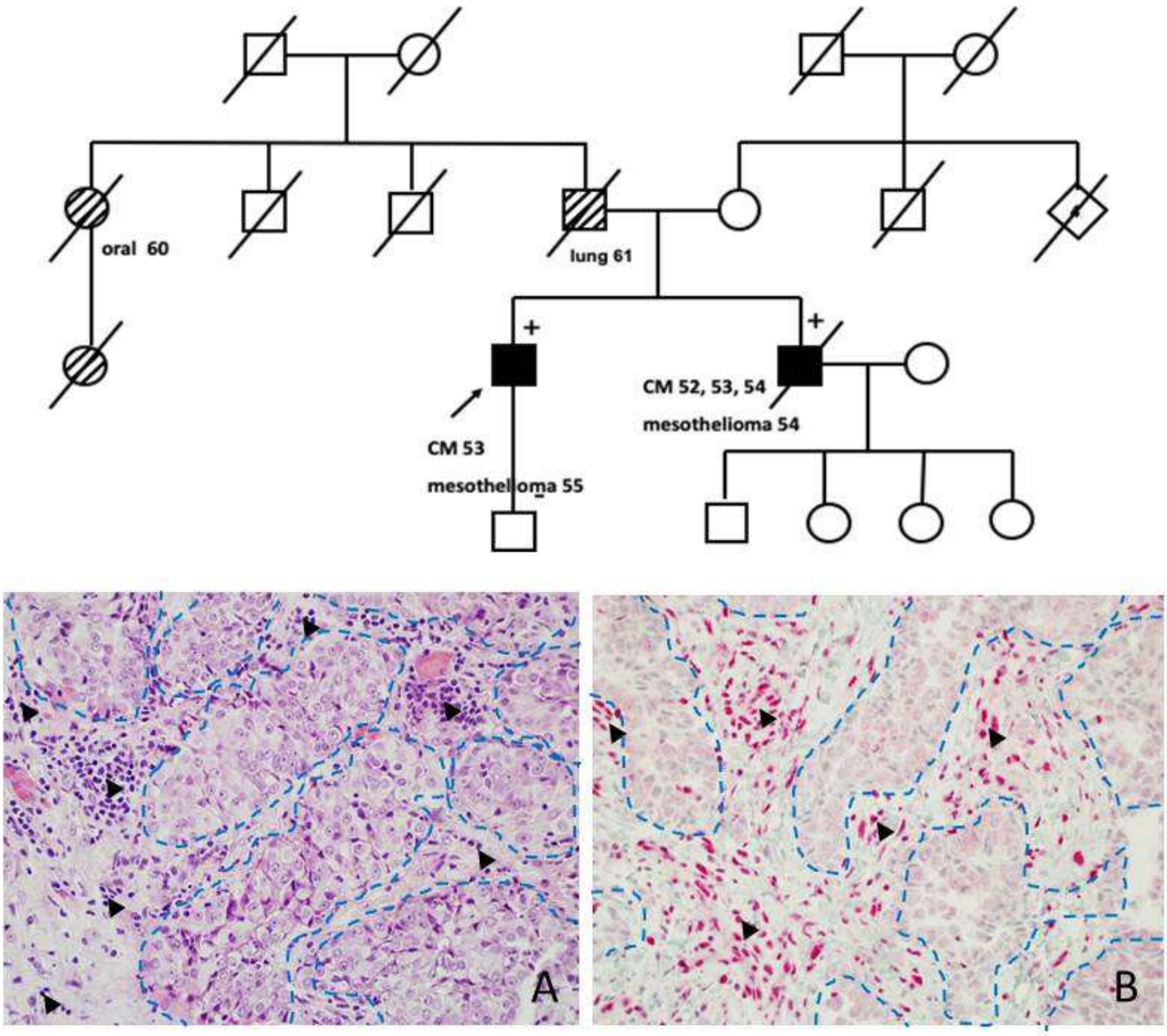

Figure S2. Pedigree diagram from the family with the rare c.799_800delCA *BAP1* germline variant. The proband developed CM and was diagnosed with mesothelioma during mutation-specific follow-up. His brother had previously developed 3 CMs and mesothelioma. (**A**) Haematoxylin and Eosin stained section of mesothelioma from a pleural biopsy (magnification ×40) from the proband's brother carrying the c. 799_800delCA *BAP1* germline variant. Within the dotted blue outline are neoplastic cells; the black arrow heads point to tumor infiltrating lymphocytes. (**B**) Section from the mesothelioma in (A) (magnification ×40) immunostained for BAP1 protein. Nuclear loss of BAP1 expression is seen in neoplastic cells within the dotted lines while the non-neoplastic lymphoid cell nuclei which serve as a control (showing no loss of BAP1 expression) stain in red (black arrow heads).Dark symbol: cutaneous melanoma (CM). Cancer type and age at diagnosis is indicated under each symbol. The black arrow in the pedigree points to the proband.

© 2019 by the authors. Licensee MDPI, Basel, Switzerland. This article is an open access article distributed under the terms and conditions of the Creative Commons Attribution (CC BY) license (http://creativecommons.org/licenses/by/4.0/).
